# The Organization, Implementation, and Functioning of Dengue Surveillance in India—A Systematic Scoping Review

**DOI:** 10.3390/ijerph16040661

**Published:** 2019-02-24

**Authors:** Eva Pilot, Vasileios Nittas, Gudlavalleti Venkata S Murthy

**Affiliations:** 1 Department of Health, Ethics and Society, Care and Public Health Research Institute (CAPHRI), Faculty of Health, Medicine and Life Sciences, Maastricht University, 6229HA Maastricht, The Netherlands; v.nittas@alumni.maastrichtuniversity.nl; 2 Maastricht Centre for Global Health, Maastricht University, Peter Debyeplein 1, 6229HA Maastricht, The Netherlands; 3 Public Health Foundation India, Indian Institute of Public Health Hyderabad, Telangana 500033, India; murthy.gvs@iiphh.org; 4 London School of Hygiene and Tropical Medicine, London WC1E 7HT, UK

**Keywords:** dengue, surveillance, India, infection, systematic scoping review, health information, public health, vector-borne diseases

## Abstract

Dengue´s re-emerging epidemiology poses a major global health threat. In India, dengue contributes significantly to the global communicable disease burden, and has been declared highly endemic. This study aims to identify and critically appraise India’s dengue surveillance system. We conducted a systematic literature review, searching Medline, Web of Sciences, Global Health, and Indian Journals. We conducted a narrative synthesis and thematic analysis. Eighteen studies fulfilled eligibility. Organizationally, most studies referred to the National Vector Borne Disease Control Programme, primarily responsible for overall vector and disease control, as well as the Integrated Disease Surveillance Programme, responsible for reporting, outbreak identification, and integration. Surveillance implementation was mostly framed as passive, sentinel, and hospital-based. Reporting varies from weekly to monthly, flowing from primary healthcare centres to district and national authorities. Dengue confirmation is only recognized if conducted with government-distributed MAC-ELISA tests. The surveillance system predominantly relies on public reporting units. In terms of functioning, current surveillance seems to have improved dengue reporting as well the system’s detection capacities. Emergency and outbreak responses are often described as timely; however, they are challenged by underreporting, weak data reliability, lack of private reporting, and system fragmentation. Concluding, India’s dengue surveillance structure remains weak. Efforts to create an infrastructure of communication, cooperation, and integration are evident, however, not achieved yet.

## 1. Introduction

Rapidly increasing globalization trends, intensifying trade, climatic changes, and continuously expanding travel patterns constitute a critical web of major global health threats [1,2]. The geographical expansion of tropical vectors, capable of carrying and transmitting outbreak-prone diseases, confirms that the occurrence of epidemics in previously non-endemic regions exceeds estimations. Global statistics reveal that nearly one-fourth of the world’s annual mortality is causally related to emerging and re-emerging diseases, with low- and middle-income countries bearing a disproportional amount of the burden [3]. In the past decades, arbovirus infection has re-emerged as an epidemic disease of global concern [4].

Dengue, an acute viral infection with potentially lethal consequences, has been declared one of the most significant and prevalent arthropod-borne communicable diseases. Dengue has been declared prevalent in more than 100 tropical and subtropical countries, accountable for accelerating trends of global morbidity and mortality. Responsible for an average of 50 million annual infections, dengue is estimated to pose a major health threat to more than 2.5 billion individuals [5]. Its gradual post-World War II re-emergence was primarily characterized by rapid geographical spread, increasing incidence rates, and regular outbreaks, assumed to be causally related to enhanced shipping trade, rising population numbers, and urbanization [6]. Clinical observations have identified a broad range of disease manifestations, ranging from mild asymptomatic flu-like symptoms to severe and often fatal complications, also known as dengue haemorrhagic fever and dengue shock syndrome [7].

India, home to 1.3 billion, constitutes a significant contributor to the global communicable disease burden. Although India’s economic and infrastructural development has been enormous, large health and social inequalities, marked by disproportionate disparities between extreme poverty and wealth, remain inadequately addressed [8]. Dengue’s emergence has exposed Indian public health authorities to severe public health challenges. In 2015, the Ministry of Health and Family Welfare (MoHFW) reported approximately 21,000 new dengue infections [9]. Research suggests that the number of dengue cases is underestimated [9]. The subcontinent has been declared highly endemic, causing regular outbreaks of all serotypes across the country. With urban environments being the preferred habitat of dengue vectors, India’s rapidly expanding cities had a considerable impact on the viruses’ spread [7]. The country´s public healthcare system is structured across various, hierarchically organized administrative levels. Public health initiatives and healthcare services operate on national, state, district, and local scales, creating a highly complex administrative web. Understanding the position and functioning of public health surveillance within India´s complex healthcare structure is crucial to estimate the burden of dengue and develop adequate responses taking into account the public and private healthcare system. 

Aims of the systematic scoping review: This study systematically reviews existing scientific knowledge on national, as well as regional dengue surveillance in India. Simultaneously, it aims to identify and critically appraise the organizational, functional, and implementation-related aspects of current dengue surveillance initiatives, contributing towards prospective improvements in timely detection and effective response. Its core objective is a better understanding of existing structures, processes, gaps and good practice, ultimately facilitating India´s public capacities for mitigating the growing burden of dengue and other vector-borne infections. 

## 2. Materials and Methods

### 2.1. Definition, Sources, and Study Identification

As public health remains a premature, but growing field in India, most of the existing research evidence addresses vector control, as well as medical and sero-epidemiological surveillance approaches, neglecting a more holistic public health focus. Aiming to reduce this gap, this study restricts dengue surveillance to its public health dimension, excluding all epidemiological, vector control, entomological, or predominantly clinical studies. For that purpose, public health surveillance is defined as the “continuous, systematic collection, analysis and interpretation of health-related data, needed for the planning, implementation, and evaluation of public health practice” [10].

Due to the missing comprehensive synthesis of existing scientific knowledge on India´s dengue surveillance initiatives, a systematic scoping review of scientific literature is considered appropriate to summarize and disseminate research findings and identify research gaps and to provide recommendations for future research [11]. The review was performed according the Preferred Reporting Items for Systematic Reviews and Meta-Analyses (PRISMA) guidelines, excluding quality assessment and quality of evidence strength [12]. 

We comprehensively searched the following four data bases: Medline, Web of Sciences, Global Health, and Indian Journals. Medline was selected for its comprehensive and broad medical scope, including more than 12 million articles. Global Health offers a narrower, however, more specific scope, with a core focus in international health and entailing a strong communicable disease component. Web of Science was primarily chosen to complement Medline, offering a wide base interdisciplinary and less clinical citations. Finally, Indian Journals was added to capture regional and context-specific literature, which might not be included in international Journals. We additionally searched the reference lists of all included studies, aiming to capture literature that might not have been covered by the electronic databases.

Keyword searches were limited to titles and abstracts, grouped into three thematic components. The first reflected the review´s geographic focus, the second addressed dengue, and the third covered surveillance. As outlined in supplementary Table S1, all utilized terms were piloted and adjusted to the syntax of each database, aiming to adequately and fully reflect the study´s research focus. To achieve maximum sensitivity and capturing all relevant literature, data-based specific Medical Subject Headings (MeSH) terms and subject headings were complemented by equivalent free-text keywords, including truncations and wildcards. The geographic component was simply covered by using the term “India”. The surveillance component was reflected by terms that cover several variants of public health surveillance, including “population surveillance” and “sentinel and syndromic surveillance”, as well as database-specific related terms such as “disease notification”, “infection”, and “disease control”. The term “monitoring” only yielded additional potentially valuable results in Web of Science and Indian Journals and was therefore excluded from the other two databases. The dengue component was covered by all identified medical terms used to express the disease. Acknowledging that fever is the main reported symptom, in many cases used interchangeably for dengue, the terms “acute fever” and “acute undifferentiated fever” were added to increase search sensitivity. The search words and MeSH terms are provided in supplementary Table S1.

### 2.2. Screening Procedures and Study Selection

Our search identified publications containing at least one keyword of each of the three components. It total, the four databases, Medline, Global Health, Web of Science, and Indian Journals yielded 1258 articles, covering the years of 1946, 1973 and all subsequent years until April 2017. The chosen period is purposively large, aiming to increase the searche’s sensitivity and comprehensively capture any potentially relevant publications. Therefore we always chose the earliest available data range of each searched database. We retrieved 278 articles from Medline, 340 from Global Health, 480 from Web of Science, and 160 from Indian Journals. Among these, 301 were excluded after initial duplication check. A total of 957 publications entered a comprehensive screening process, undergoing eligibility checks according to a set of pre-defined inclusion and exclusion criteria. Those are listed in supplementary Table S2.

Study identification was followed by a rigorous three-step inclusion process, conducted by two reviewers independently. The first stage entailed the title and abstract screening of all retrieved publications, applying the exclusion and inclusion criteria and excluding those deemed thematically irrelevant. Inconsistencies were resolved concordantly. In the second step, all remaining studies where assessed for eligibility on full text basis, re-applying the eligibility criteria. Ensuring comprehensiveness, all reference lists of the final selected 18 articles where manually screened, aiming to capture relevant literature that might have not been captured by the search. 

### 2.3. Data Extraction and Synthesis 

Data extraction and synthesis followed a systematic and standardized process. We created a matrix, in which we entered all relevant information, allowing for consistency and transparency. The collected variables were classified in five thematic areas. Firstly, we extracted information that summarizes and offers a general descriptive picture of each study, including the utilized methods, sample sizes, covered time periods and geographic focus. The next three themes reflected the study´s key focus and extracted information on the organization, implementation and functioning of dengue surveillance. Organizational information targeted primarily descriptive and normative information, such as involved surveillance institutions and the level of governance. Implementation refers to actual application of surveillance processes, including applied data sources, reporting procedures and the types of existing surveillance. Functioning predominantly captured information on the gaps, strengths, and weaknesses of existing approaches. Finally, the last theme of research primarily intended to identify future research needs. The extracted data were synthesized narratively. The design and heterogeneity of included studies did not allow for meta-analytic or other quantitative approaches. The entire search and screening procedure as well as data extraction was conducted by two independent researchers, followed by regular result comparison, discussions and commonly agreed results. 

## 3. Results

**Figure 1:**
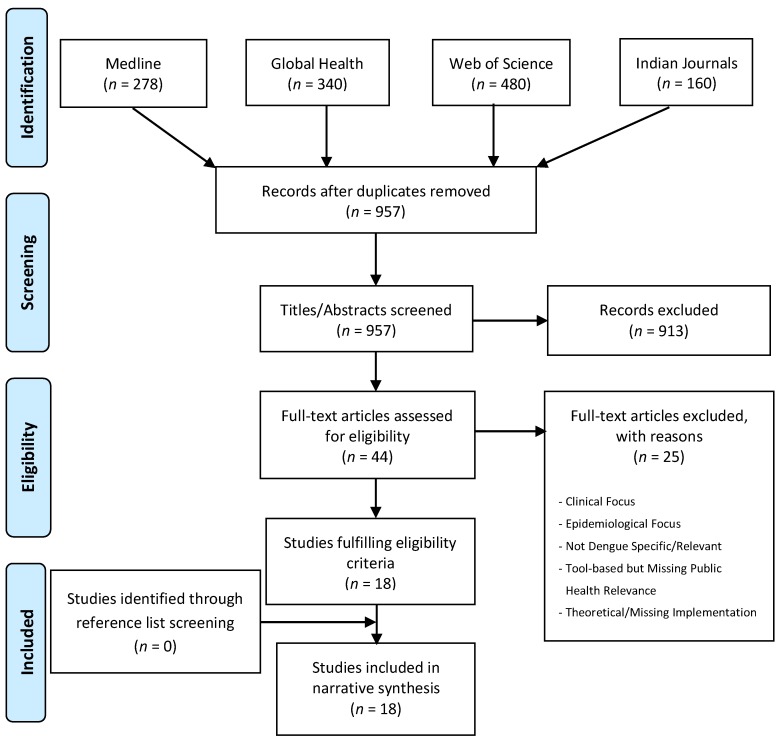
Systematic literature review PRISMA flow diagram.

Our searches generated 957 citations, of which 913 were excluded at title and abstract screening. Full-text appraisal was conducted on 44 studies, of which 25 did not fulfil eligibility. Final inclusion was deemed appropriate for 18 studies. Figure 1 provides a PRISMA flow diagram of the study identification and selection process, including reasons for exclusions. 

### 3.1. Summary of Selected Scientific Literature

Table 1 provides a list of the 18 eligible and included studies, attaching each to an ID number. For practical purposes, the following results paragraphs refer to all identified articles using those IDs. None of the included studies was yielded by the India Journal Database, which potentially indicates the low prioritization of public health focused surveillance, especially dengue-related, in Indian scientific literature. The initially identified articles (*n* = 160), dealing with dengue, were mainly clinical, epidemiological, or vaccine-oriented, lacking public health elements. Accordingly they were excluded. To avoid further fragmentation and confusion, institutes which were renamed or merged but were still referred to with the old names in the articles were amended with the up-to-date information.

Table S3 in the supplementary provides an overview of the literature review. It contains a brief summary of organization, implementation, and functioning of the selected dengue surveillance literature in India. 

### 3.2. Scale and Scope—Literature Characteristics

The sample of included studies ranges from 2004 to 2017, with most of the publications (*n* = 10) occurring between 2014 and 2015. The majority of identified studies predominantly describe national dengue surveillance structures (*n* = 11), including those studies that concurrently focus on various regions and administrative levels across India. Six of the reviewed studies primarily applied a regional focus, covering the states of Tamil Nadu, Delhi, and Gujarat, as well as the districts of Kottayam (Kerala) and Guntur (Andhra Pradesh). Thus, the geographic distribution of reviewed studies ranges from the country´s southern parts, over Tamil Nadu and up to the south-eastern coast, covering the western regions close to the neighbouring state of Pakistan and finally reaching the capital region of Delhi.

The most commonly referred research aim (*n* = 6) targeted the review and description of India´s existing surveillance structures and dengue control approaches, followed by a predominant focus on the identification and understanding of dengue´s evolution, expansion and burden (*n* = 5). Four studies aimed to address the key challenges, as well as weaknesses of the country´s dengue surveillance system, while three studies targeted the piloting and evaluation of new, innovative approaches, such as Geographic Information System (GIS) and emergency-data based initiatives. Within those studies, two focused on data availability and two on cost estimations. All included studies (*n* = 18) yielded information on the functioning of India´s dengue surveillance system, including strengths, weaknesses and required action. Most studies provided information on the system´s actual implementation (*n* = 16), while a substantial number of articles offered valuable descriptions of organizational elements (*n* = 14). None of the reviewed manuscripts covered the aspect of urban versus rural surveillance as a main point.

A substantial proportion of studies adopted mixed methodologies (*n* = 9), with most combining literature reviews and secondary data analysis or synthesis (*n* = 8), followed by the combination of literature review and case studies (*n* = 1). In fact, most reviews (*n* = 12) were entirely based or included a review component. Data analysis or synthesis was the second most common approach (*n* = 9) and was followed by case studies (*n* = 4).

### 3.3. Organization of Dengue Surveillance

As the majority of studies clearly indicated, India’s dengue surveillance falls under the auspices of the Ministry of Health Family Welfare (MoHFW), and is primarily operationalized, organized and controlled by the National Vector Borne Disease Control Programme (NVBDCP) (ID, 2,4,8,9,10,11,12,13,16,17), as well as the Integrated Disease Surveillance Programme (IDSP) (ID 4,5,10,11,12,13,18). A crucial organizational change, as well as turning point for the country´s national dengue surveillance, was the expansion and renaming of the previous antimalarial program to what today constitutes the NVBDCP, aiming to establish a national-level responsibility for other crucial vector-borne infectious diseases (ID 4), including dengue. In fact, following the recommendations by the Planning Commission, all major and endemic vector-borne diseases are now kept under the NVBDCP´s auspices and organizational control (ID 9). A core organizational responsibility includes the framing of national dengue guidelines and policies, aiming to guide the implementations of state-level surveillance and control strategies (ID 8). Finally, the umbrella agency aims to prevent and control dengue´s rapidly re-emerging expansion across regions and at different time intervals, maintaining systematic epidemiological data (ID 9,11). 

**Figure 2:**
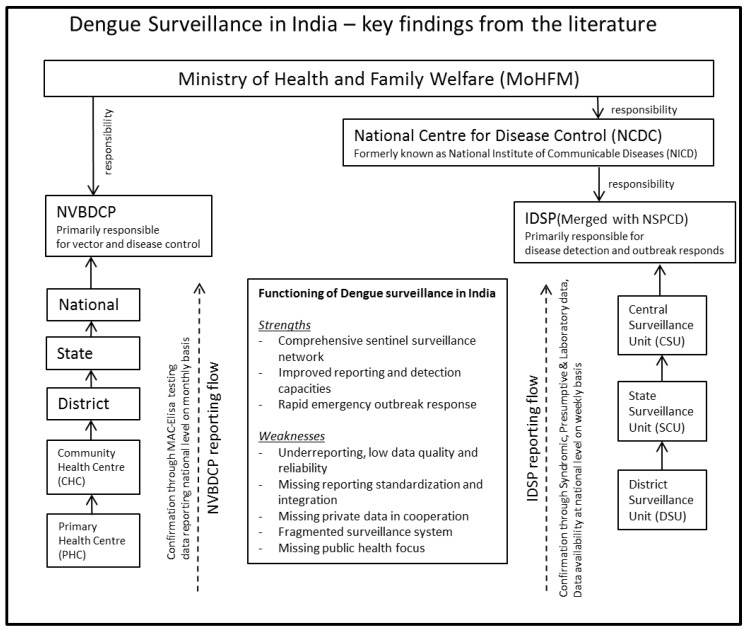
Key findings from the literature review. National Vector Borne Disease Control Programme (NVBDCP); Integrated Disease Surveillance Programme (IDSP); National Surveillance Programme for Communicable Diseases (NSPCD).

A further organizational turning point was the 2006 administrative and financial merger of the National Surveillance Program for Communicable Diseases (NSPCD) and the IDSP. The IDSP, playing a significant role in India´s dengue surveillance, is a decentralized state based surveillance system, targeting a few outbreak prone diseases of public health importance (ID 4). Organizationally, it falls under the oversight of the National Centre for Disease Control (NCDC) (ID 9,14), formally known as the National Institute for Communicable Diseases (NICD) (ID 4). The system, launched in 2004 and initially supported by the World Bank and WHO, is set up to improve overall surveillance procedures and to specifically enhance laboratory networking and quality assurance, as well as to review case definitions and facilitate the integration of inefficiently and vertically operating disease control programs (ID 4,5). Although it was originally conceptualized for a few reporting units, the IDSP gradually expanded, intending to connect the whole public health reporting system within districts, while offering the opportunity of merging resources and databases for improved operations, better planning and increased effectiveness of infectious disease control (ID 4). Figure 2 provides an overview of organization structure as represented in the literature including strength and weaknesses of dengue surveillance.

Additionally, the literature underlines a few other organizations, of secondary role but clearly linked to dengue surveillance. One of these is the Central Bureau of Health Intelligence (CBHI), which is a national nodal institute under the control of MoHFW, holding the responsibility of providing health profiles for various diseases including dengue (ID 12). A second one is DengueNet, which is an Internet-based central data management system of global scope, used for information-sharing on dengue fever (ID 11). Finally, the GVK Emergency Management Research Institute (GVK EMRI) is collecting syndromic health information, generated through the 108 emergency medical services (ID 18). 

### 3.4. Implementation of Dengue Surveillance 

The majority of included studies (*n* = 11) described the implementation of India´s dengue surveillance as sentinel, hospital-based, and predominantly passive, while a few highlight the system´s reliance on laboratory surveillance (ID 2,3,4,8,9,10,11,12,13,14,16). Only one study reviewed the potential of syndromic surveillance for dengue (ID 18) and one case study is focusing on passive voluntary dengue surveillance (ID 3). The following paragraphs provide a literature summary on the actual implementation of dengue surveillance, divided into NVBDCP, IDSP, and a paragraph on case studies.

### 3.5. National Vector-Borne Disease Control Program

Although NVBDCP data confirm that dengue is established and endemic across India, an exclusive dengue surveillance system does not exist (ID 7,11). Instead, dengue surveillance is embedded within the NVBDCP’s sentinel surveillance network, consisting of 500 sentinel hospitals and 15 referral laboratories (ID 2,) across India. The number of reporting units increased overtime as previous studies describe networks of 137 sentinel hospitals and 13 referral laboratories (ID 9,10), while others mention 347 sentinel hospitals and 14 referral labs (ID 8), respectively. The difference in numbers of reporting units (hospitals and laboratories) is not always directly linked to the publication year. In fact, the presence of sentinel hospitals varies substantially between states (ID 2). Bihar, the third most populated state, has only seven hospitals, in strong contrast to the National Capital Territory Delhi, which ranks 18th in terms of population size and has 33 hospitals (ID 2). NVBDCP´s sentinel hospitals and referral labs confirm dengue using the IgM antibody capture enzyme-linked immunosorbent assay (MAC-ELISA) kit, developed by the National Institute of Virology (NIV) in Pune (ID 2,16). The central government determines the distribution of MAC ELISA kits annually, based on recorded cases of the previous year. Information flows from Primary Health Care Centres (PHC) and Community Health Care Centres (CHCs) to a district medical officer, to be finally forwarded to state-level NVBDCP authorities. Dengue confirmation with MAC ELISA can lead to delays in reporting. As almost the entire sentinel system is public, dengue surveillance relies primarily on public actors’ data. Neither NVBDCP implementation nor the reporting of dengue is legally mandatory, leaving implementation and reporting to rely upon the capacity and willingness of states (ID 2,9). The number of reporting units correlates with the number of reported cases, indicating much higher incidence numbers in well-represented regions (ID 2). During inter-endemic periods, reporting to the NVBDCP can reach monthly intervals, while narrowing down to daily reports during outbreak seasons (ID 4,9). Ad hoc assistance for outbreak investigation and control exists (ID4) and is provided by the federal government of India and coordinated by the district medical officer (ID 9). Often the earliest attention to dengue outbreaks and thus, initiation of response, is triggered via media reporting (ID 4).

### 3.6. Integrated Disease Surveillance Program

IDSP consists of a parallel administrative hierarchy, operating on three levels. The highest is the Central Surveillance Unit (CSU) on national level, followed by the State Surveillance Units (SSU) on sub-national level and District Surveillance Units (DSU) on a regional level (ID 13). IDSP primarily depends on three mechanisms for reporting of infectious endemic-prone diseases, such as dengue. The first reporting channel is based on syndromic diagnosis and occurs through syndromic forms (S forms), completed by nurses and field workers on the availability of symptoms. The second mechanism relies on medical doctor diagnosis, without laboratory confirmation and reported through presumptive forms (P forms). The third is based on laboratory diagnosis occurs through laboratory forms (L-forms) (ID 12). Similar to the NVBDCP, IDSP reporting primarily depends on public actors and occurs on weekly intervals, including disease alert reports (ID 4,11,13). The NVBDCP and IDSP reporting remain largely fragmented, despite efforts to integrate and ultimately merge data flow. The literature indicates that NVBDCP´s district Malaria officers have been urged to actively share their reports, as well as closely cooperate with IDSP authorities, aiming to streamline procedures and reduce inefficiencies (ID 4). The following paragraphs provide a selection on regional case studies provided in the literature, with the first three containing examples of innovative, yet not scaled up, dengue surveillance approaches and the last three offering regional insights in state dengue surveillance reporting programs. 

### 3.7. Case Studies from the Literature 

The first case study reported in the literature was set up in 1999–2001 by the central district medical office and aimed to test district-level surveillance in Kottayam, Kerala (ID3). The passive voluntary surveillance set up was based on postcards and included public, as well as private reporting facilities for 14 selected diseases, including dengue. Reporting occurred through medical doctors and without the requirement of laboratory confirmation, avoiding reporting delays and eliminating excuses for reporting dismissal. Reporting was followed by analysis, targeting the detection of clusters. Despite confidentiality issues and missing etiological underpinnings, the system was assessed as very sensitive to early outbreak signals and disease clustering, potentially beneficial for timely detection and response (ID 3).

A second case study aimed to quantify the spatio-temporal epidemiology of dengue in large urban areas using GIS systems and focusing on Delhi, the most dengue-affected region in India. The use of GIS-based analysis identified core dengue surveillance deficiencies, despite the existence of an extensive reporting network, concluding the true burden estimation is impossible in most parts of the country (ID 16). In fact, the use of GIS for analysing epidemiological data provides a promising opportunity to implementation and evaluation of dengue surveillance.

A third case study aimed at assessing the contribution of routinely collected health information data from the Emergency Medical Service (EMS) to improve infectious disease surveillance and early warning using dengue as an illustrative example. The study was based on a mixed method approach, including qualitative interviews and EMS data analysis for Guntur District in Andhra Pradesh (ID 18). The system for early warning based on emergency data (SEED) analysed incoming Acute Undifferentiated Fever (AUF) chief complaint calls, received at Andhra Pradesh´s state dispatch centre, aiming for early dengue outbreak identification. In fact, analysis indicated promising results, suggesting that outbreaks were detected before any other form of reporting, and recommended the integration of SEED into IDSP reporting system (ID 18).

A further case study, conducted in the state of Gujarat aimed to compare IDSP laboratory reports to literature-based dengue rates across India, evaluating the regional implementation of IDSP. Analysis states that despite remaining technical, administrative and financial challenges, as well as differences to national reports, IDSP has increased Gujarat´s dengue identification capacities, with L-forms providing the most accurate picture (ID 12). A secondary data analysis conducted in Tamil Nadu confirms the fragmented reporting between NVBDCP and IDSP, suggesting that the latter´s data indicate lower accuracy, failing to capture the actual dengue burden (ID 13). In a final case study that targets the same region, the authors describe the implementation of NVBDCP as beneficial, increasing Tamil Nadu´s detection power and dengue outbreak response. Tamil Nadu´s distinctive element is the establishment of a Public Health Department, which does not exist in any other state. Thus, surveillance data flow from designated laboratories to the Director of Public Health in the capital region of Chennai. Additionally, Tamil Nadu is the only state in India with reported efforts to provide dengue-related training for public as well as private practitioners (ID 17).

### 3.8. Functioning of Dengue Surveillance 

#### 3.8.1. Strengths

The system strength reported in the literature reflects the well-established and geographically spread network of NVBDCP reporting facilities and sentinel surveillance units across the country (ID 4,10,17). High endemicity areas, such as Delhi, are covered by a large sentinel network, which in turn entails sufficient coverage of public hospitals that facilitate dengue detection (ID 16). Despite underreporting, figures indicate that the network has contributed to the surveillance system´s case detection power, further facilitated by increased dengue sensitivity among healthcare providers (ID 8,10,17). In addition, being connected to designated laboratories, detection power is often accompanied by improved and timely outbreak response (ID 17). Equally promising is the infrastructure of IDSP, especially for achieving integration of vertically and inefficiently operating programs (ID 3). Even though it is not used to its full capacities yet, one study suggests that IDSP has contributed to strengthening laboratory networks, quality assurance of dengue diagnosis and reviewed dengue case definitions (ID 5). As already outlined in the case studies above, regional evaluations, such as in the state of Gujarat, suggest that IDSP contributes to epidemiological investigations and thus, to better surveillance (ID 12). Rapid emergency and outbreak response is reported as strength for various states in India (ID 9). Finally, the development and wide application of diagnostic test kits and techniques like MAC ELISA, provided for free to labs by the Government of India (GoI), indicates promising results and benefits overall dengue surveillance (ID 11). 

#### 3.8.2. Weaknesses

The most commonly reported weaknesses of India´s NVBDCP dengue surveillance constitute severe underreporting, partially attributable to missing active surveillance components, as well as to lack of legal enforcement (ID 2,4,5,8,9,11). In fact, it is suggested that NVBDCP captures less than 0.5% of the annually occurring dengue cases across India (ID 8). That is reflected in the system´s overall low sensitivity, capturing only severe cases, accumulated during inter-endemic periods (ID 2,10). Consequentially, that is linked to weak outbreak prediction and detection (ID 9). Additional weaknesses include the inefficient distribution of MAC ELISA test kits, as well as the low ratio of laboratory confirmed cases (ID 2,9). Furthermore, NVBDCP´s dengue reporting and case inclusion criteria seem to lack standardization and coherence, causing differences between state and national reports, as well as variation in reporting cohesion between states (ID 8). 

Underreporting, attached to inconsistencies, irregularities and inaccurate data is also a challenged faced by IDSP (ID 5,11,12,13). Regional evaluations, such as in Tamil Nadu, indicate that the IDSP fails to capture actual dengue incidence (ID 13). Frequent changes in the program´s functioning and reporting formats as well as procedures have adversely affected its overall performance (ID 4). Limited reporting units and missing integration within other vertical programs, including the NBDCP, as well as within existing health care structures deems it virtually ineffective for controlling dengue and other infectious diseases (ID 4). “Incrementally adding new programmes will not solve the fundamental systematic deficiency” (ID 4). Thus, IDSP´s full potential and expected benefits are yet to be realized (ID 4,12).

Moving beyond individual programs and focusing on the dengue surveillance system´s overall weaknesses, studies report high fragmentation, missing integration and cooperation between key agencies such as the IDSP and NVBDCP, and missing active and public health surveillance, as well as low outbreak prediction capacities (ID 4,6,7,15)

The Central Bureau of Health Intelligence (CBHI) reports less than half the case rate for all types of diseases compared to the IDSP in the state of Gujarat (ID 12). Despite the population’s high dependency on the private health sector, private involvement in dengue reporting and control remains low to non-existent (ID 4,10,11,12). Finally, a broader criticism targets the system´s strong reliance on out of pocket expenditure, which ultimately enhances the already large health inequalities (ID 4). National law is generally not enforcing the reporting notifiable diseases as the currently operating public health act of 1897 has never been amended or updated; with the revised draft is still pending in 2011 (ID 4). 

#### 3.8.3. Needs

A commonly addressed need to enhance dengue surveillance across India is the establishment and strengthening of a long-term, comprehensive public health approach, fostering permanent and active surveillance, across states and regions (ID 1,4,7,11,15,16). Surveillance networks involving all agencies (NVBDCP, NSPCD, IDSP) need to be further strengthened, as efforts remain restricted to endemic periods, while the system mainly relies on informal sources, such as media reporting, for outbreak identification (ID 4,17). Dengue surveillance needs to be addressed with a systems approach; generating reliable information and valid data on dengue is the obvious first step (ID 12,13,6). The essential for “accurate IDSP alert reporting through better collection, collation, compilation and validation of data” is highlighted (ID 13). Additionally, it is essential to enhance early warning of dengue outbreaks, timely data availability, as well as the exploration of routine health data and additional data sources for disease surveillance (ID 18). Finally, dengue awareness and a better understanding of its manifestation needs to be established within health care networks, as well as the whole public as argued by John et al. (ID 2).

### 3.9. Future Research Components for Improved Dengue Surveillance

Future research needs to establish dengue as a priority in India, facilitating the development of action recommendations for establishing active surveillance, prioritizing vector control, as well as enhancing public awareness and prevention approaches (ID 9). Research on the integration, without duplication, of the so far inefficient vertical programs into a real-time comprehensive and interlinked surveillance system is essential (ID 4). 

In addition, national-level studies estimating true disease burden and its geographical expansion as well as examining its relationship with environmental, climatic and human-induced determinants are essential for improved dengue control (ID 5, 13). Large, population-based studies on key incidence determinants and the identification of vulnerable populations would fill important knowledge gaps on dengue´s epidemiology, emergency and complexity. Literature suggests that this would be further facilitated by dedicated and dengue-specialized research laboratories (ID 10,11). There is a need for further studies including retrospective designs to refine economic burden of dengue (ID 8). Finally, enabling open access to IDSP data would potentially facilitate stakeholder involvement, especially from the public, as well as the academic sector (ID 12), potentially contributing to enhanced data collection and analysis (ID 4). “As health information in India still demonstrates significant reliability and validity problems, further investment in health data research, to further identify the underlying reasons and develop and enhance a framework for an integrated health information system, is needed” (ID 18). 

## 4. Discussion

This review aimed to systematically identify and narratively synthesize published literature on the organization, implementation and functioning of public health dengue surveillance in India. WHO´s global health strategy aims to reduce mortality and morbidity attributable to dengue by 50% and 25% respectively within the next three years [28]. Enhanced outbreak predictions, efficient case detection, rapid outbreak responses and integrated, as well as coordinated surveillance approaches are perceived as crucial elements for successfully controlling dengue´s re-emergence [28]. Achieving those targets additionally requires a comprehensive understanding of existing surveillance structures, strengths and weaknesses, primarily in highly endemic regions. Despite clear evidence that endemic dengue is present throughout the country, this review highlights a range of remaining surveillance weaknesses across India. Overall, existing infrastructure indicates promising characteristics; however, the gap between what is required to adequately counteract dengue´s re-emergence and what currently exists is substantial.

Organizationally, the literature predominantly focuses on two key authorities, the NVBDCP, functioning as the umbrella organization responsible for dengue surveillance and control, and the IDSP, which is primarily described as aiming to integrate and enhance existing structures. The two agencies, although connected, operate largely independently according to the reviewed literature. Several studies suggest strong and continuing fragmentation between the NVBDCP and IDSP, which ultimately leads to inefficiencies, duplication, and potential waste of valuable resources. The major challenges of public health surveillance overall in India lies in linking information from the several separately operating health care systems and in bridging the knowledge and information gap among the various operating health programmes and stakeholders [29]. Undeniably, India requires a restructured health information system that is integrated and reliable [29], [30]. This is also depicted by the national health policy 2017 which highlights strengthening the overall health system. In particular, the health surveillance system needs to be strengthened and a federated integrated health information architecture needs to be established according to the 2017 health policy [30]. The development of a national health information network should be based on standards that ensure the inter-operability among all stakeholders in the healthcare sector according to the Planning Commission of India [29]. This is connected to the findings of the general fragmentation within the dengue surveillance system and the need to overcome the fragmentation by strengthening the interaction and linking of surveillance programmes. The incorporation of private data for dengue reporting remains weak and often non-existent [29,31]. Under both authorities (NVBDCP and IDSP), implementation is structured hierarchically, with information flowing from primary levels, to district state and national-level authorities. In addition to multiple levels, complexity seems to be added by multiple and parallel operating reporting channels. This leads to separate, not joined, reports at the respective government levels with duplication and selective reporting risks. The IDSP´s three-tier reporting system, with data flowing from nurses, doctors and laboratory staff individually has the potential of capturing cases that might never reach the lab or hospital, ultimately enhancing sensitivity. Whether the required analytical capacities are in place to exploit all these incoming data is questionable and requires further research. The question that arises here is how and to what extent those hierarchies impact systems effectiveness and efficiency. If not analysed comprehensively, the system risks to simply produce and never use large amounts of syndromic and presumptive reports.

Moving towards surveillance functioning and concrete strengths and weaknesses, the system´s flaws are primarily reflected in the quality, availability and reporting of dengue surveillance data, across responsible authorities. Arguably, dengue places an extra high burden on healthcare systems, as it is difficult to identify whether an infected patient is at higher risk to develop severe complications, which often leads to precautious but unnecessary hospitalizations [32]. Despite the limited resources of India’s public health system [33] it is crucial to have a functioning surveillance system that produces reliable data. Nonetheless, the literature suggests that missing case standardization and uniform reporting rules, exclusion of the very dominant private healthcare industry, and system fragmentation lead to severe underreporting and low data quality. Specific case studies, such as in the state of Tamil Nadu, highlighting that reported dengue figures deviate from actual incidence rates, reflect the system’s severe underreporting challenge. One of the largest and potentially most challenging weaknesses is the missing integration of private healthcare facilities, which in many regions cover the health needs of most of the population. If the numbers of reported cases are not reliable, how can we expect timely, sensitive and adequate emergency and outbreak responses? These observations are in line with the broader epidemiological literature, suggesting that India remains one of the countries with greatest dengue burden uncertainty [34]. Failure to capture true incidence has wider consequences, such as severely limited capacities to evaluate vaccination effectiveness, as the outcomes, such as reported cases or hospitalization are highly unreliable [34]. Although identifying the true dengue burden is a distant or even utopian aim, re-adjusting the system in ways that will facilitate data quality, reporting procedures, and estimation across geographic locations and socio-economic backgrounds seems to be the most urgently required action to reduce India´s vulnerability to dengue and other emerging and re-emerging diseases. 

Acknowledging that NVBDCP emerged from an exclusively antimalarial function to a broader vector control programme, one could ask to what extent the previous malaria-specialized infrastructure has been adapted and emerged to fully encompass the needs for dengue and other vector-borne diseases. Vector-borne infections contribute to approximately 10% of the global communicable disease burden. Considering limited resources and an overlap in vectors, transmission routes, and control mechanisms, integrating surveillance and control across diseases such as dengue, malaria, lymphatic filariasis, and the currently emerging Zika is considered highly desirable [35]. Unfortunately, the IDSP, which was implemented under the overarching aim of streamlining and integrating various vertically operating services indicates little true integration of dengue surveillance and despite his high potential, it is often described as ineffective and weak in achieving its core aims. Our findings are consistent with overall surveillance weaknesses across India, including inefficient and parallel running programs, unreliable statistics, underreporting and missing private involvement [36].

Despite clear weaknesses, the literature highlighted certain system strengths that should not be neglected, as they can play a crucial role in learning and future improvements. Although the surveillance system remains insufficient, progress is undoubtable. The sentinel surveillance network has considerably expanded, leading to gradual reporting improvements and better connectivity across regions. Innovative and promising surveillance approaches, even if not fully implemented yet, such as the piloting of GIS systems, the use of alternative reporting procedures and the use of EMS data for early warning are signs of progress and advancement. All those approaches remain small-scale and in test phases, depending on political will, adequate resources, and an appropriate infrastructure for further expansion. It remains to be seen whether the system will actually allow for their integration and scale-up. A final important consideration to be made is the fact the existing system provides indications that it can be effective; however, it requires an adequate supporting infrastructure to facilitate efficient functioning, as well as targeted efforts to reduce the apparent inefficiencies in fragmentation, reporting and data quality. 

Future research can be a key facilitator of progress, acting as a tool to fill knowledge gaps and as an incentive for innovation. Further research on the actual health, social and financial burden of dengue is required. Large-scale trials, specifically targeted at how to effectively and efficiently integrate existing surveillance approaches, across vector-borne diseases, are urgently required [35]. Finally, the literature indicated little focus on the rural/urban division of dengue surveillance. Although largely neglected in the scientific literature we have analysed, geographic variations, and primarily rural-urban dynamics are crucial for the development of an integrated and equitable surveillance system. In fact, urban infrastructures, commonly operating under regional, independent authorities are often stuck to outdated public health programs and fragmented infrastructure, significantly limiting their capacities for effective infectious disease surveillance and control [8]. Further research on geographic variations, dynamics and inequalities would certainly facilitate a stronger, more integrated, equitable system. 

### Limitations

The study has a few limitations to be considered when interpreting the results. Firstly, due to pragmatic reasons and resource limitations, inclusion was only allowed for studies written in English, potentially excluding relevant studies in various regional Indian and other languages. For the same reason, the review included the systematic search of only four search databases. Although the selected databases have been carefully chosen to yield a sensitive search output, we acknowledge that a broader number of databases might have yielded additional studies. Thirdly, grey literature and government reports were excluded.

## 5. Conclusions

This review summarizes and underlines the poor organization, implementation, and functioning of public health surveillance of dengue in India. Although efforts to create an infrastructure of communication, cooperation and integration are evident, those aims have not been achieved yet. With other vector-borne diseases such as Zika gradually emerging and with dengue’s burden constantly growing, India can only ensure a healthier future by removing boundaries, allowing integration, and facilitating comprehensive and inclusive reporting of public, as well as private public health data. 

Dengue needs to be perceived as an emerging and serious public health threat, requiring comprehensive, specialized, and simultaneously integrated approaches. 

## Figures and Tables

**Table 1:** List of selected publications on organization, implementation and functioning of dengue surveillance in India.

ID	Selected Studies	Author (Year Publication) [Reference Number]
(1)	Chikungunya fever, falciparum malaria, dengue fever, Japanese encephalitis ... are we listening to the warning signs for public health in India?	Bhargava and Chatterjee (2007) [13]
(2)	Combating dengue in India: Challenges and strategies	Daude and Mazumdar (2016) [14]
(3)	Communicable disease monitored by disease surveillance in Kottayam district, Kerala state, India	John et al. (2004) [15]
(4)	Continuing challenge of infectious diseases in India	John et al. (2011) [8]
(5)	Current perspectives on the spread of dengue in India	Gupta and Ballani (2014) [16]
(6)	Dengue epidemiology in selected endemic countries: Factors influencing expansion factors as estimates of underreporting	Toan et al. (2015) [17]
(7)	Dengue surveillance poor in India	Bagcchi (2015) [9]
(8)	Economic and disease burden of dengue illness in India	Shepard et al. (2014) [18]
(9)	Emergence of dengue problem in India—A public health challenge	Sharma et al. (2014) [19]
(10)	Fifty years of dengue in India	Chakravarti et al. (2012) [7]
(11)	Fight against dengue in India: Progress and challenges	Gupta and Reddy (2013) [20]
(12)	Infectious disease burden in Gujarat (2005–2011): Comparison of selected infectious disease rates with India	Iyer et al. (2014) [21]
(13)	Outbreak of dengue in Tamil Nadu, India	Chandran and Azeez (2015) [22]
(14)	Overcoming data limitations: design of multi-component study for estimating the economic burden of dengue in India	Halasa et al. (2011) [23]
(15)	Urgent need for a permanent dengue surveillance system in India	Sivagnaname et al. (2012) [24]
(16)	The spread of dengue in an endemic urban milieu—The case of Delhi, India	Telle et al. (2015) [25]
(17)	Laboratory-based dengue fever surveillance in Tamil Nadu, India	Victor et al. (2006) [26]
(18)	Towards sustainable public health surveillance in India: Using routinely collected electronic emergency medical service data for early warning of infectious diseases	Pilot et al. (2017) [27]

## Supplementary Materials

The following are available online at https://www.mdpi.com/1660-4601/16/4/661/s1, Table S1: Search word and MeSH term overview linked to databases used for review, Table S2: Inclusion and exclusion criteria of literature review, Table S3: The literature review summary of organization, implementation and functioning of dengue surveillance in India.
