# Supplementary material for: The Organization, Implementation, and Functioning of Dengue Surveillance in India—A Systematic Scoping Review
Source: Int J Environ Res Public Health. 2019 Feb 24;16(4):661. doi: 10.3390/ijerph16040661 (PMC6407027; doi:10.3390/ijerph16040661)
Supplement: Supplementary file 1 [file ijerph-16-00661-s001.zip › ijerph-428552-suppl/Supplementary table 3.pdf]

Table S3. The literature review summary of organization, implementation and functioning of dengue surveillance in India.

ID	Aim of Study & Method used	Specific Region Sample size	Time period	Organization & Actors	Surveillance Type & Implementation Details	Functioning - Strengths & Weaknesses & Need
(1)	**Aim:** Outlines challenges of vector borne disease control in India<br><br>**Method**: Essay Review, Case Studies	India overall<br><br>No Sample	2005-2006	X	Dengue government strategy is based on malaria vector control. The public health response consisted of dispatching medical teams and medical camps in times of outbreaks. Thermal fogging machines were used, although guidelines emphasize limited effect. Community received false sense of security, attached highly visible action. Political statement: eradicate mosquitos in 3 years	**Weakness:** Inadequate data and unreliable and delayed reporting. **Weakness:** Neglect of the basic requirements of health; poor political support for health; a weak public health capacity; centralized programme for control based on selective interventions, and poorly-planned development projects -creating conditions ideal for the outbreak of disease. **Weakness:** Vector control not effective as based on malaria vector that has different breeding habits. None of our national health programme has reliable data on the magnitude of the disease. **Need:** For comprehensive & long-term public health response
(2)	**Aim**: Discussing existing surveillance challenges and identifying potential strategies for surveillance enhancement<br><br>**Method:** Narrative review, case studies & data analysis	India overall<br><br>No Sample	2006-14	NVBDCP network	**Sentinel/ hospital-based passive surveillance** Each states wealth determining factor of participation in surveillance system Reporting of confirmed IgM MAC ELISA cases through NVBDCP linked network of 500 sentinel	**Weakness:** Surveillance system likely to only capture most severe cases, within poorest population. Limited official data available, fueling debate about actual numbers **Weakness:** Non-accurate and inefficient MAC ELISA distribution system by the central government **Weakness:** Low sensitivity of sentinel surveillance during inter-endemic

					hospitals and 15 apex referral labs<br><br>Central government determines distribution of MAC ELISA kits annually based on recorded cases of previous years<br>Predominantly public data reported<br>Number of recorded cases correlated to number of available sentinel hospitals in a state<br><br>Reporting not mandatory by law. Implementation by will and capacity of states	periods (underreporting)<br>**Weakness:** Lacking involvement of private hospitals (only 1 of 95 in Andra Pradesh, Delhi, and Tamil Nadu)<br>**Weakness:** Additional underreporting via missing of active surveillance components & strong variation between states and districts, as well as non-mandatory reporting nature of dengue<br>**Need:** For enhanced awareness of disease and symptoms for population as well as doctors (public and private)
(3)	**Aim:** Testing a new district disease surveillance model<br>**Method:** Pilot experiment for district level surveillance project including of: a) training of officers and paramedical staff and b) implementation of postcard reply system for infectious disease surveillance c) analyzing of incoming reports to detect outbreaks	Kottayam district, Kerala<br><br>Sample: 2427 reported cases	1999-2001	Central district medical office Nodal officer for district level disease surveillance	**Passive and voluntary surveillance**<br>Participating public and private facilities reporting cases of 14 diseases (including fever with bleeding tendencies in order to include DHF) via business card postcards<br>Data are analyzed by the district medical office for the identification of clusters (outbreaks)<br>Doctors report diseases on day of clinical diagnose and do not wait for lab confirmation – avoiding	**Strength:** Classified as effective for obtaining early signals and disease clustering, especially for unusual diseases, however, active ingredients not elaborated in further detail<br>**Strength:** IDSP offers promising infrastructure to streamline vertical programs (not fully used yet)<br>**Weakness:** Confidentiality issues, as postcard content can be read by anyone<br>**Weakness:** Inability to conduct epidemiological and aetiological investigations on many of the reported diseases due to lacking personnel skills and laboratory support<br>**Weakness:** Lab access was meagre, relatively expensive and under-used

					delay in reporting & preventing excuse for not reporting a case Director of Health Services denied information on prevalence of dengue in state	when available
(4)	**Aim:** Reviewing current state of India´s response to infectious diseases **Method:** Literature review & secondary data analysis	India overall No Sample	2011	Principal Actors: NVBDC, under Ministry of Health and Family Welfare MoHFW Additional Actor: IDSP (since 2004) Field Support Actors: National Institute for Communicable Diseases Notifiable Diseases are generally not reported as the current operating public health act of 1897 has not been amended.	**Passive, sentinel hospital-based surveillance** Programs operating vertically & non-integrated, while diseases left out of these programs mostly neglected Surveillance reports occur primarily through the NVBDCP and IDSP networks, however, not in real time Reporting primarily conducted by public health care actors Media report outbreak (sensationalize) State Department (deny or underestimate) Ad-hoc assistance for outbreak investigations and control exists The entire system is strongly based on out of pocket expenditure & commercial health care,	**Strength:** Good network of facilities across the country, including sentinel units **Weakness:** High fragmentation of healthcare system, leading to inefficiencies **Weakness:** Underreporting & missing case-based surveillance (also visible on international level, as WHO SEARO – India data often not available) **Weakness:** Missing private involvement in reporting (IDSP), lacking resources, weak ownership & low reporting quality **Weakness:** Missing integration of IDSP and NVBDCP **Weakness:** Big gaps in surveillance and response. Data not available in timely manner during crisis **Weakness:** Missing reform. The Public Health Act from 1897 has not been amended & is still circulating in parliament, while notifiable diseases are generally not reported. **Need:** India needs to rethink and revise health policy to broaden the agenda of disease control. A Public Health approach is needed, as only one state

					which is increasing health inequality	(Tamil Nadu) has a professional Director of Public Health.
(5)	**Aim:** Reviewing the factors for the changing epidemiology of Dengue in India **Method:** Literature review & summary of secondary data	India overall No Sample	1996-2014	IDSP	Implementation of IDSP promising but severely lacking	**Strength:** IDSP contributed to strengthening laboratory networks, quality assurance and case definition reviewing **Weakness:** Dengue surveillance very limited & reporting to the central government is not mandatory **Weakness:** IDSP indicates epidemiological gaps & underreporting, as well as low capacities of identifying true dengue burden
(6)	**Aim:** a) Compare the national and local data reported from routine surveillance systems & studies b) identify critical factors, able to influence the calculation of the expansion factors as an estimate of the extent of underreporting **Method:** Systematic Review	Selected SEARO, WPRO and PAHO countries No Sample	2000-2013	X	X	**Weakness:** No active surveillance data in India – indicating reporting weaknesses
(7)	**Aim:** Awareness raising of reporting-surveillance challenges for Dengue **Method**: Opinion Paper (short review)	India overall No Sample	No specific time span	X	**Passive surveillance** No exclusive surveillance system in place in India for dengue	**Weakness:** Substantial underreporting, as estimations indicate that fever cases in India could be 282 times higher than the known numbers **Weakness**: Missing public health department and the much-needed public health surveillance **Need:** For strong public health surveillance, dedicated on Dengue, as that is then only way to identify true burden

(8)	**Aim**: Estimating disease burden and direct medical cost of Dengue in India<br>**Method:** National Retrospective study	Various states and cities across the country<br><br>No Sample	2006-2012	NVBDCP Network	**Selective sentinel surveillance (Hospitals and Labs)**<br>Sentinel Surveillance Network with 347 sites and 14 Apex Referral labs, under the control of NVBDCP (predominantly public)<br>Dengue reporting and reporting inclusion criteria not standardized, causing differences between state and national reports	**Strength:** Sentinel network seems to have improved reporting, despite remaining underreporting<br>**Weakness**: Severe underreporting & missing of cases outside of the selected sentinel surveillance network<br>**Weakness**: NVBDCP estimated to capture only 0.35% of annual actually occurring clinically diagnosed dengue cases across India<br>**Weakness**: Missing reporting cohesion between states
(9)	**Aim:** Review the present situation for Dengue prevention and control in India<br>**Method:** Systematic Review (including grey literature)	Various states and cities across the country<br>No Sample	X	Principal Actor: NVBDCP<br><br>Support Actors: Government of India<br>National Centre for Disease Control (referral lab)	**Passive, sentinel hospitals-based surveillance**<br>NVBDCP is responsible for framing national dengue guidelines and policies, as well as guiding the implementation of program strategies at state level- overlooking all important vector borne diseases<br>System includes 137 sentinel hospitals and several referral labs for monitoring and confirming dengue cases (Predominantly public)<br>Monthly dengue cases are reported to the directorate of NVBDCP. Reports	**Strength:** Rapid emergency/outbreak response<br>**Weakness**: Limited active surveillance. System relies almost fully on medical officers, who are responsible for distinguishing dengue from other febrile illnesses<br>**Weakness**: Severe underreporting leads to weak capacities to detect outbreaks and contain early epidemics<br>**Weakness**: Lack of general knowledge: less than 50% of respondents knew measures to reduce mosquitoes (Karnataka), while less than 1% was aware that dengue is transmitted by mosquitos<br>**Weakness**: Only 5% of clinically diagnosed cases sent for lab confirmation during outbreaks.<br>**Need:** Creating a specific dengue/chikungunya initiative would

					occur daily during outbreaks<br>First point of contact during a suspected outbreak is the district medical officer<br>Outbreak support during endemic periods (in endemic states) is provided by the Government of India<br>Emphasis lies on emergency control of outbreaks- committees initiated at district or municipal level (resources and guidance for standardized management)	attract research and enable control
(10)	**Aim:** Provide an overview of available data on the epidemiology of Dengue to improve the understanding of its evolution<br>**Method:** Literature Review and summary of secondary data	India overall<br><br>No Sample	1965-2010	NVBDCP<br>IDSP<br>State governments	**Passive, sentinel hospital-based surveillance**<br>The system includes primary health and community health centers (hospitals > 30 beds), primarily including public actors<br>The System relies on disease notification by health care professionals, who are required to report all suspected cases<br>Reporting of lab confirmed Dengue cases (with MAC ELISA,	**Strength:** During the last years there have been more reported cases, attributable to newly established endemicity and increased sensitivity among healthcare professionals<br>**Strength:** Widespread reporting coverage (geographically)<br>**Weakness**: Low surveillance sensitivity during inter-endemic periods (due to passive approach)<br>**Weakness**: 90% of health service is private, while 40 % of dengue treatment is private. At the same time, the private sector is under-represented in dengue surveillance<br>**Weakness**: Large number pf dengue publications- mainly focus on small

					developed by National Institute of Virology, Pune) flows from primary and community health centers to NVBDCP's district medical officers, who forward those to NVBDCP's state offices	case series usually from few neighboring hospitals.
(11)	**Aim:** Review and outline various aspects of dengue control in India- focusing on the challenges and achieved progresses<br>**Method:** Narrative review	India overall<br><br>No Sample	X	State government/ Government of India<br>NVBDCP<br>IDSP<br>DengueNet is an internet based central data management system (Global, not India specific), used for information sharing	**Passive, sentinel hospital-based surveillance**<br>The NVBDCP responsible for monitoring and coordination across states, while commodities, funding and technical assistance is provided by the government of India and official responsibility remains with the state governments<br>System relies primarily on public actors, while the private sector remains unregulated	**Strength:** Initial stage advances in use of GIS for identifying high risk areas and populations & initial developments of new diagnostic technique (rapid solid-phase)<br>**Strength:** MAC ELISA tests provided for free to labs by GoI immunochromatographic technique with promising results<br>**Weakness**: Underreporting & reporting discrepancies, causing a misleading epidemiological picture due to 1) misdiagnoses 2) occurrence of silent cases 3) low awareness of patients and not seeking medical attention 4) major deaths taking place at home in rural areas without noticed 5) unregulated private healthcare 6) poor medical and diagnostic facilities 7) missing legal requirements<br>**Weakness**: Surveillance restricted to endemic regions<br>**Weakness**: Missing active surveillance- which would be beneficial in identifying risk-prone areas and vulnerable populations (in combination with remote sensing tech. and GIS)<br>**Weakness**: Weak diagnosis due to

						cost-ineffectiveness of MAC ELISA and use of various other tests (with varying sensitivities)<br>**Need:** Urgent need for permanent & active surveillance.
(12)	**Aim:** Comparing IDSP laboratory reports of various diseases (including Dengue) to rates given in the literature from other parts of India<br>**Method:** Literature search and secondary data analysis	Gujarat State<br><br>No Sample	2005-2011	IDSP<br>NVBDCP<br>Nodal institute for health intelligence Central Bureau of health Intelligence (CBHI)	**Passive, sentinel hospital-based surveillance**<br>IDSP reporting occurs through three mechanisms, a) syndromic b) presumptive and c) laboratory confirmed (S, P, L Forms), with L forms providing most accurate picture System predominantly captures urban cases	**Strength:** In Gujarat, IDSP has increased the number of reports, although numbers remain inconsistent with national reports. It seems that IDSP contributes to epidemiological investigations and thus, to better surveillance **Weakness**: IDSP is weak due to technical, managerial and financial challenges<br>**Weakness**: Reporting inconsistencies & irregularities
(13)	**Aim:** Identify correlations between climatic/power supply factors and dengue & develop prediction model (of infections)<br>**Method:** Statistical analysis of secondary data on rainfall, power supply and dengue cases	Tamil Nadu State<br><br>No Sample	2010-2012 - monthly data on rainfall, power supply and dengue cases	IDSP<br>NVBDCP	**Passive, sentinel hospital-based surveillance**<br>Weekly disease alert reports of IDSP launched by Central Surveillance Unit (CSU), State Surveillance Unit (SSU) and District Surveillance Unit (DSU)<br>Annual count of dengue from NVBDCP network	**Weakness**: NVBDCP data provided higher accuracy for future predictions than IDSP data, suggesting that IDSP fails to capture actual dengue epidemiology<br>**Weakness**: Poor reporting and data accuracy, including due to weak data collection strategies<br>**Need:** IDSP requires further attention in collection, compilation and validation of data
(14)	**Aim:** Presenting an approach to estimating the cost of dengue illness in India<br>**Method:** Mixed approach of cost estimation, including	India Overall	X	Ministry of Health and Family Welfare<br>National Centre for Disease Control	**Passive, sentinel, laboratory, hospital-based surveillance**<br>System includes 330	**Weakness**: Underreporting<br>**Weakness**: Lack of availability of systematically compiled data<br>**Need:** A broader study is needed to estimate the overall economic burden

	retro- and prospective data collection & statistical analysis from multiple sources	No Sample		Directorate General of Health Services	reporting facilities and 14 apex labs,<br>System designed to monitor outbreaks and guide outbreak responses (national, regional and state level)	**Need:** Analyzing hospital records can adjust surveillance data for possible under-reporting and misdiagnosis of dengue
(15)	**Aim:** Present a normative view of how a good surveillance system in India should look like?<br>**Method:** Review	India overall<br><br>No Sample	X	X	X	**Weakness**: Current used entomological indices are mainly based on immature survey – neither qualitative nor informative regarding forecasting dengue outbreak<br><br>**Weaknesses:** Systems on monitoring and notification of symptomatic cases have low sensitivity and are not capable of detecting low or sporadic transmission<br><br>**Need:** Better virological surveillance during the inter-epidemic periods<br><br>**Need:** Active lab based surveillance and better understanding of epidemiology for effective prevention.<br><br>**Need:** Permanent dengue surveillance system, across and in all states
(16)	**Aim:** Quantify spatio-temporal epidemiology of Dengue in large cities using GIS, aiming to identify socio-economic risk factors<br>**Method:** Secondary data analysis, using GIS	Delhi<br><br>No Sample	2008-2010	NVBDCP network	**Passive, sentinel hospital-based surveillance**<br>Public and private sentinel surveillance hospitals, part of the NVBDCP network,	**Strength:** Good coverage of public hospitals, including a very large network in Delhi<br>**Weakness:** Biases due to surveillance deficiencies- as in most parts of India Dengue cannot be correctly reported in urban milieu

					reporting MAC ELISA confirmed cases<br>Delhi: Sentinel Network of 33 public and 3 private hospitals (2010) with all public hospitals with more than 100 beds included in system. Delhi is the most Dengue affected city in India<br>Kerala: 10 sentinel hospitals<br>Tamil Nadu: 13 sentinel hospitals<br>West Bengal: 10 sentinel hospitals<br>Bihar: 1 Sentinel hospital	**Need:** Surveillance is needed in each state to forecast an outbreak.<br>Effective dengue control program need information on occurrence of infection and disease
(17)	**Aim:** Strengthen the Dengue surveillance network in India and prevent major dengue outbreaks<br>**Method:** Review and discussion paper	Tamil Nadu State<br><br>No Sample	1998-2006	NVBDCP	**Laboratory based Surveillance**<br>System under umbrella of NVBDCP– with reporting sites up to primary health care level<br>Reporting flows from designated laboratories (performing MAC Elisa) to the Directorate of Public Health in Chennai<br>Training occurs for public health cadre PHCs, district entomologist, health inspectors and lab assistants<br>Training is also reached	**Strength:** Tamil Nadu has experiences increased detection power of new cases & rapid outbreak response<br>**Need:** Network of all agencies needs to be further strengthened

					out to private practitioners, with a special focus on dengue symptoms	
(18)	**Aim:** Assess routinely collected health information data from Emergency Medical Services for improved infectious disease surveillance and early warning capacity.<br><br>**Method:** mixed method approach; semi-structured interviews and EMS fever data analysis	Guntur, Andhra Pradesh<br><br>Sample: 1,595 fever emergency calls	January-September 2010	GVK Emergency Management Research Institute – EMS operating system Andhra Pradesh, India<br><br>IDSP	**Syndromic surveillance based on AUF – Acute Undifferentiated Fever**<br><br>System for Early Warning Based on Emergency Data (SEED)<br><br>Incoming emergency calls captured in the AP state dispatch center in Hyderabad are assessed based on chief complaint approach<br><br>Retrospective and Prospective analysis of fever cluster outbreak was tested. Dengue fever outbreak was detected before any media reporting.<br>Possible integration of SEED into IDSP at state surveillance unit for enhanced early warning of infectious disease – dengue – outbreak.	**Strength:** Cost effective complementary system for enhancing early warning of infectious diseases (example dengue). Limitation in surveillance system in India early warning - SEED can enhance.<br><br>**Weakness:**<br>Just symptom (AUF) used & no lab confirmation.<br>Just severe cases are detected due to health seeking behavior of EMS use.<br><br>**Need:** SEED just tested in AP, India. Further development in automated system needed.<br><br>**Need:** Capacity building and leadership is needed for PHS in India.<br>**Need:** further investment in health data research

* NVBDCP-National Vector Borne Disease Control Programme (http://nvbdcp.gov.in/). * IDSP-National Integrated Disease Surveillance Programme (http://idsp.nic.in/). *x- No content reported.
