# Supplementary material for: The Organization, Implementation, and Functioning of Dengue Surveillance in India—A Systematic Scoping Review
Source: Int J Environ Res Public Health. 2019 Feb 24;16(4):661. doi: 10.3390/ijerph16040661 (PMC6407027; doi:10.3390/ijerph16040661)
Supplement: Supplementary file 1 [file ijerph-16-00661-s001.zip › ijerph-428552-suppl/supplementary.pdf]

Table S 1. Search word and MeSH term overview linked to databases used for reveiw.

**Database 1: Medline** / Time: from 1946 till 2017 / Link: https://ovidsp.tx.ovid.com/sp-3.23.1b/ovidweb.cgi
India (MeSH) or India*
AND
Population Surveillance (MeSH) or Public Health Surveillance/ (Subheading) or Sentinel Surveillance (Subheading) or communicable disease control (MeSH) or disease notification (Subheading) or infection control (Subheading) surveillance (keyword) "syndromic surveillance" (keyword) or "symptom-based surveillance" (keyword)
AND
Dengue (MeSH) or severe Dengue (Subheading) or Dengue virus (MeSH) or DENV (key word) "Dengue fever" (keyword) or DF (keyword) or "Dengue haemorrhagic fever" (keyword) or DHF (keyword) or "Dengue shock syndrome" (keyword) or DSS (keyword) or "acute fever" (keyword) or "acute undifferentiated fever" (keyword)
**Database 2: Global Health** / Time: from 1973 till 2017 / Link: https://ovidsp.tx.ovid.com/sp-3.23.1b/ovidweb.cgi
India (Subject Heading) and India*
AND
Surveillance (Subject Heading) or sentinel surveillance (Subject Heading) or syndromic surveillance (Subject Heading) or disease control (Subject Heading) or infection control (Subject Heading) surveillance (keyword) or control (keyword) or management (keyword)
AND
Dengue (Subject Heading) or Dengue haemorrhagic fever (Subject Heading) or Dengue shock syndrome (Subject Heading) or Dengue virus (Subject Heading) or DENV (key word) "Dengue fever" (keyword) or DF (keyword) or "Dengue haemorrhagic fever" (keyword) or DHF (keyword) or "Dengue shock syndrome" (keyword) or DSS (keyword) "acute fever" (keyword) or "acute undifferentiated fever" (keyword)
**Database 3: Web of Science** / Time: all years / Link: https://apps.webofknowledge.com
India*
AND
Population Surveillance or Public Health Surveillance or Sentinel Surveillance or disease surveillance or surveillance or disease control or disease notification or infection control or control or management or syndromic surveillance or symptom-based surveillance or monitor*
AND
Dengue or Dengue fever Dengue virus or DENV (MeSH) or haemorrhagic fever or Dengue shock syndrome or DF or DSS or DHF or acute fever or acute undifferentiated fever
**Database 4: Indian Journals** / Time: all years / Link: http://www.indianjournals.com/ijor.aspx
India*
AND
Surveillance or control or management or monitor*
AND
Dengue or "Dengue fever" or DENV or DF or "Dengue haemorrhagic fever" or DHF or "Dengue shock syndrome" or DSS or "acute fever" or "acute undifferentiated fever"

Table S 2. Inclusion and Exclusion Criteria of literature review.

Inclusion	Exclusion
1. Fully written in English	1. Not fully written in English
2. Scientific & Peer-reviewed articles	2. Grey literature (government reports, project report, etc.)
3. Focusing on India	3. Primarily addressing entomological surveillance
4. Published between 1946 and April 2017	4. Primarily addressing sero-epidemiological and virology surveillance
5. Addressing national, state, district, rural or municipal Dengue surveillance	5. Only mentioning public health surveillance in the conclusion and recommendation sections
6. Entailing a public health surveillance focus	
